# Supplementary material for: A Novel RHS1 Locus in Rice Attributes Seed-Pod Shattering by the Regulation of Endogenous S-Nitrosothiols
Source: Int J Mol Sci. 2022 Oct 30;23(21):13225. doi: 10.3390/ijms232113225 (PMC9655508; doi:10.3390/ijms232113225)
Supplement: Supplementary file 1 [file ijms-23-13225-s001.zip › Supplementary file S1-final.pdf]

Table S1. Eight mutant gene list and their annotation

**LOC_Os ID(MSU)**	**Annotation (Description)**
LOC_Os03g31240	C2H2-type domain containing protein.
LOC_Os06g17280	PHD-finger family protein, expressed
LOC_Os10g27380	NAM protein domain containing protein.
LOC_Os10g17740	C2H2 zinc finger protein, expressed
LOC_Os04g41250	Armadillo/beta-catenin repeat family protein, putative, expressed
LOC_Os02g13890	RING/FYVE/PHD-type domain containing protein.
LOC_Os02g42370	Tyrosine protein kinase domain containing protein
LOC_Os01g02730	Similar to Receptor-like kinase
LOC_Os03g03910	Nitric oxide excess1

Table S2. Expression of *RHS1* in *A. thaliana* and rice after CySNO infiltration and itsorthologs in different plant species

	**Control**	**CySNO treatment**	**Log2 value**	**p-value**	
LOC_Os04g41250	6.41	5.69	-0.887	0.003389	Down-regulated
AT1G27910	9.87	46.55	2.23695	0.000544	Up-regulated

**Species**	**Orthologous gene**	**Putative function**
Arabidopsis	*AT1G27910*	Plant U-box 45
Brachypodium	*Bradi5g14110*	Ubiquitin-protein ligase
Maize	*GRMZM2G135629*	Ubiquitin-protein ligase
Maize	*GRMZM2G389789*	Ubiquitin-protein ligase
Rice	*LOC_Os04g41250*	Armadillo/beta-catenin repeat family protein, putative, expressed

Table S3. Primer sequences used for qRT-PCR in the study

OsNOA1	*LOC_Os02g01440*	AGAGAAGTTGGAGTTACATTGAC	ACTCTCAAATGCAGTTCACCAC
OsNIA1	*AK102178*	TCA AGG TGT GGT ACG TGG TG	CGA GGT CAT AGC CCA TCT TC
OsNIA2	*AK102363*	TGT ACC AGG TCA TCC AGT CG	CGA TGA CGT ACC ACA CCT TG
OsGSNOR	*LOC_Os02g57040*	TGTCTGGAAGGGAACAGCTT	CTTCATGCAGCAGGTCAAAC
SHAT1	*LOC_Os04g55560*	CAACCGCTACAGCAGCTGCA	GACGATGAATGCAGCGATCTTG
Sh4	*LOC_Os04g57530*	ACCAGTGCAATGACAAGTGG	GAGAGCACCTCGGAGAGC
qSH1	*LOC_Os01g62920*	ACCGGCAGTACTACCAGCAG	ACGGCGATCTCCTTGTTTAG
OsPR1a	*LOC_Os07g03710*	AGTTCGTCGAGCAGGTTATCCT	AGATTGGCCGACGAAGTTG
OsPR10b	*LOC_Os12g36850*	TGTGGAAGGTCTGCTTGGA	CACTCGTGAAGCAAAAACACA
OsNH1	*LOC_Os01g09800*	CTGATCCGGTTTCCCTCGGA	GACCTGTCATTCTCCTCCTTG
OsWRKY45	*LOC_Os05g25770*	GGACCAGGGCGATGTCACGT	TGTCCATCCATGATTCTTCGGTGA
OsJAZ1	*LOC_Os04g55920*	CCCGGAGATGCCGAT	CATACTATGCATAGAAATGGAGAC
OsJAZ5	*AK061842*	TTCACCGGTTCCTCGAGA	TGAGGTTTCTTGGGTTGTACTG
OsJAZ8	*LOC_Os09g26780*	AAAGCGACGAAAGTGCAAGT	TCGCCTGTGCTTACGATTTG
OsRSH1	*LOC_Os04g41250*	CAACACATCCATGTGCTCAA	GATTGAAGGAGCCAACCCTA
OsUBI	*Os03g0234350*	GACGGACGCACCCTGGCTGACTAC	TGCTGCCAATTACCATATACCACGAC

Table S4. Evaluation of biomass of rice seedlings exposed to nitosative stress

Treatment	WT(Dong-jin)		*rhs1*	
	Shoot length (cm)	Fresh biomass (g/plant)	Shoot length (cm)	Fresh biomass (g/plant)
CySNO (0 Mm)	4.80 ± 0.40 ^{a}	0.360 ± 0.034 ^{a}	4.78 ± 0.60 ^{c}	0.381 ± 0.010 ^{a}
CySNO (10mM)	4.40 ± 0.13 ^{ab}	0.324 ± 0.015 ^{b}	2.93 ± 0.83 ^{c}	0.276 ± 0.005 ^{c}
CySNO (50mM)	4.39 ± 0.17 ^{ab}	0.320 ± 0.008 ^{b}	2.90 ± 0.92 ^{c}	0.273 ± 0.005 ^{c}
