# Supplementary figures and images for: A Novel RHS1 Locus in Rice Attributes Seed-Pod Shattering by the Regulation of Endogenous S-Nitrosothiols
Source: Int J Mol Sci. 2022 Oct 30;23(21):13225. doi: 10.3390/ijms232113225 (PMC9655508; doi:10.3390/ijms232113225)

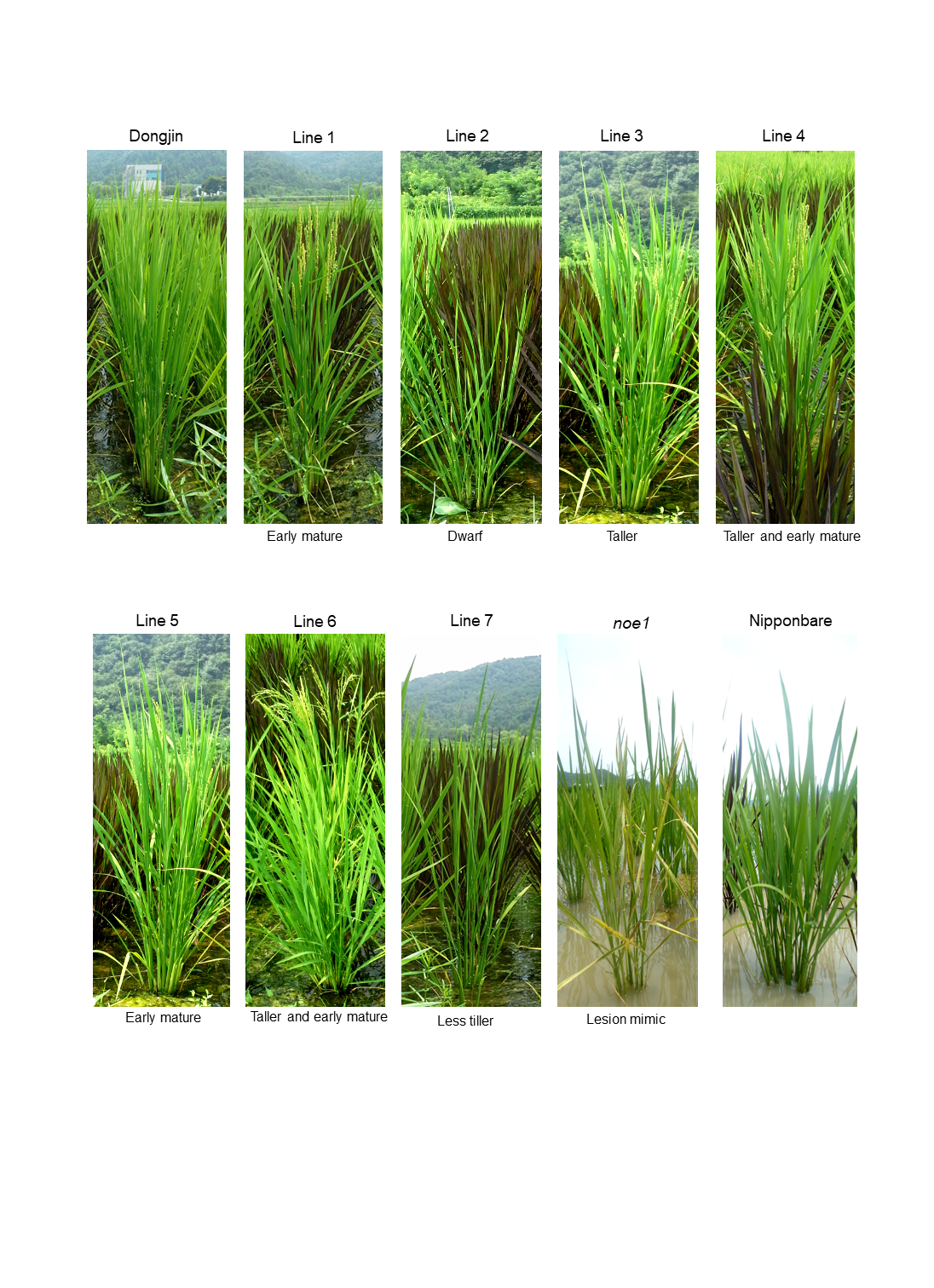

Supplement: Supplementary file 1 [file ijms-23-13225-s001.zip › Supplementary Figure S1.tif]

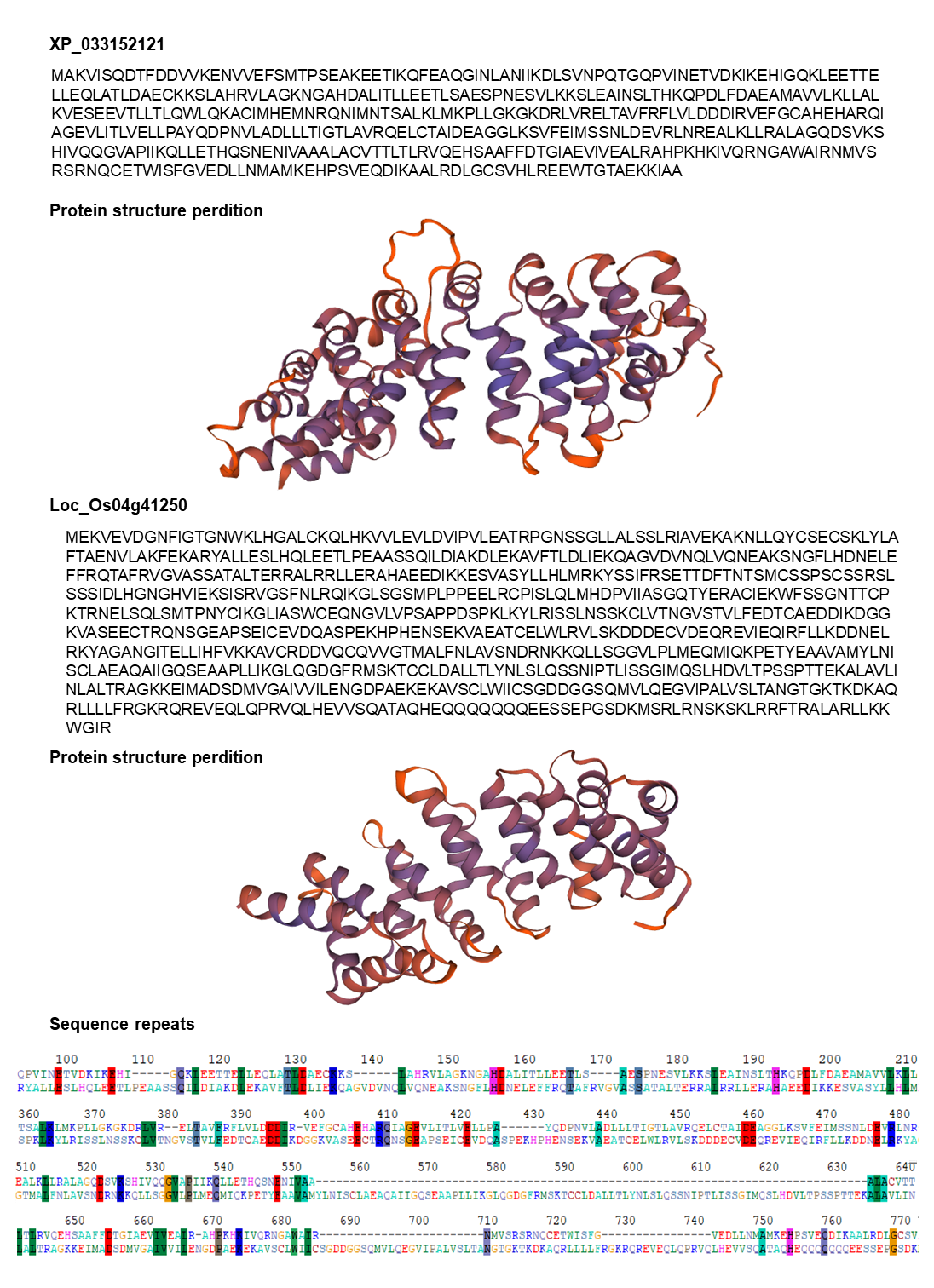

Supplement: Supplementary file 1 [file ijms-23-13225-s001.zip › Supplementary figure S2.tif]

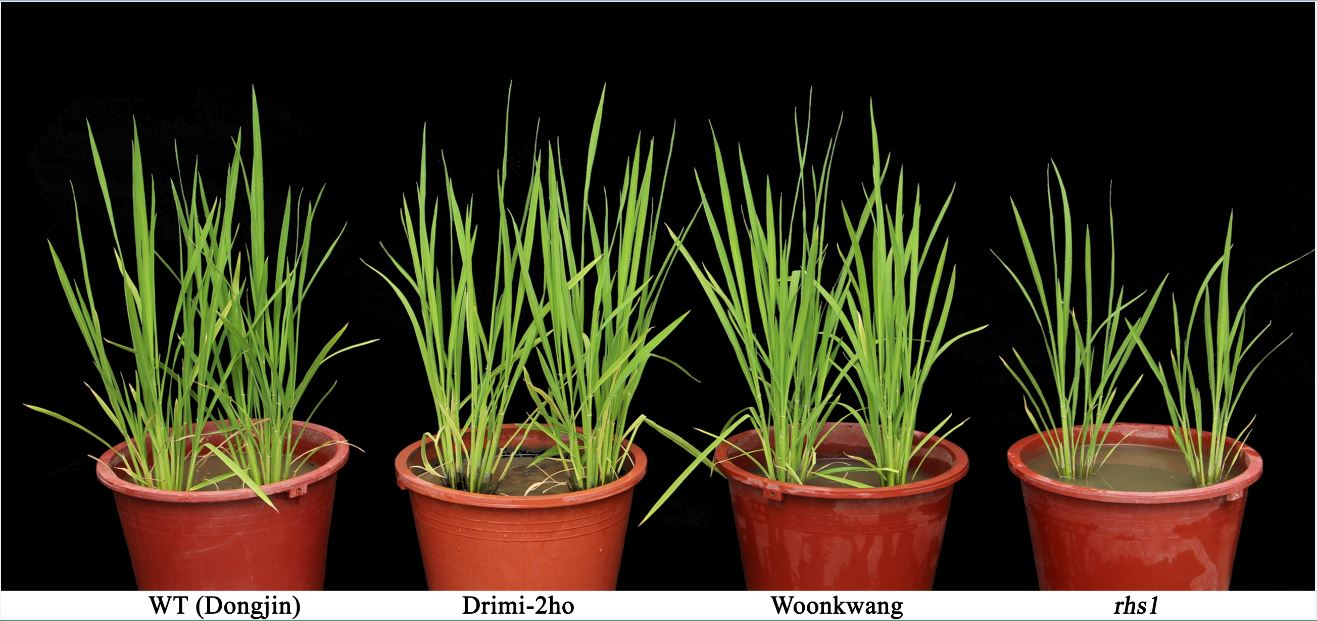

Supplement: Supplementary file 1 [file ijms-23-13225-s001.zip › Supplementary Figure S3.tif]

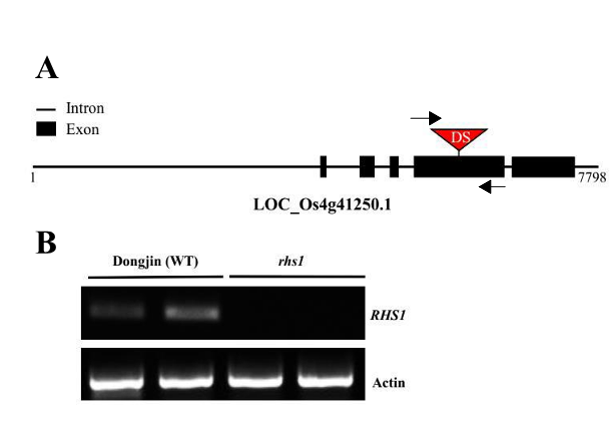

Supplement: Supplementary file 1 [file ijms-23-13225-s001.zip › Supplementary Figure S4.tiff]
